# Glutaminolysis gets the spotlight in cancer

**DOI:** 10.18632/oncotarget.14384

**Published:** 2016-12-30

**Authors:** Daniel Herranz

**Affiliations:** Institute for Cancer Genetics, Columbia University, New York, NY, USA

**Keywords:** glutaminolysis, glutamine, AML, cancer metabolism, targeted therapies

The fact that cancer cells rewire their metabolism to meet their extreme energetic and biosynthetic needs has long been known, since the seminal studies by Otto Warburg demonstrated that cancer cells heavily rely on glycolysis even in the presence of oxygen, the so-called Warburg effect. However, efforts to therapeutically target the special requirements of cancer cells have only gained traction in the last decades. In particular, beyond glycolysis and the Warburg effect, recent studies have highlighted a very prominent role for glutaminase (*GLS*) and glutamine metabolism in several types of hematological and solid tumors. Glutaminase is the rate-limiting enzyme in glutaminolysis and it is encoded by two different genes, *GLS* and *GLS2*. In turn, each of these encode for two different isoforms, kidney (KGA) and glutaminase C (GAC) for *GLS*, and liver (LGA) and glutaminase B (GAB) for GLS2. To further complicate the picture, different tissues express different levels of all these isoforms. In the current issue of Oncotarget, Matre *et al.* [1] comprehensively analyze the expression of *GLS* and *GLUD1* (which catalyzes the second reaction in the glutaminolytic pathway) in AML. Their results suggest that AML cells preferentially express the GAC isoform of GLS, with much lower expression levels of the KGA isoform. Moreover, they find that AML cases with complex cytogenetics and certain AML molecular subtypes express the highest levels of GAC, suggesting that targeting glutaminolysis in these cases could be an effective therapeutic approach. Consistently, treatment of AML cell lines and AML primary cells with specific GLS inhibitors, such as CB-839 or BPTES, results in decreased cell viability and increased apoptosis. These results further reinforce the importance of glutaminolysis in AML reported by previous studies [2, 3]. In addition, ^13^C and ^15^N-isotope labeling experiments demonstrated that inhibition of GLS with CB-839 blocks glutamine utilization and results in decreased levels, and decreased isotope labeling of glutamate, aspartate and several TCA cycle intermediates. Moreover, and in line with a previous report [4], their results suggest that targeting glutaminolysis in AML cases harboring IDH1/IDH2 mutations could be a particularly attractive therapeutic approach. Thus, CB-839 treatment promoted cell growth arrest, reduced the levels of the oncometabolite 2-HG and promoted myeloid differentiation.

This study adds up to previous literature that genetically demonstrated the therapeutic benefits of targeting glutaminase in different cancer types. Specifically, germinal loss of one *Gls* allele resulted in blunted tumor progression in a mouse model of hepatocellular carcinoma (HCC) [5]. Moreover, secondary deletion of both copies of *Gls* in already established tumors resulted in significant antileukemic effects, and highly synergistic interaction with gamma-secretase inhibitor treatment, in a mouse model of NOTCH1-induced T-cell Acute Lymphoblastic Leukemia (T-ALL) [6]. Overall, these reports support the enthusiastic efforts to target glutamine metabolism as a novel therapeutic approach in cancer therapy [7, 8].

Still, the discovery of the therapeutic benefits of targeting glutaminase in certain subtypes of AML opens new important questions. First, the metabolic routes preferred by AML subtypes that show low expression levels of glutaminase remain to be addressed. In line with this, it would also be worthy to know whether these AML cases show mutations in genes that might drive increased glycolysis, via increased PI3K/AKT or Ras signaling pathways. Moreover, complete cancer remission is unlikely to be achieved by single agent targeting of glutaminolysis, therefore, appropriate drug combination therapies should be tested in each particular cancer type that shows glutamine dependency. Finally, glutaminase inhibitors are currently being tested in clinical trials in certain solid tumors and hematological malignancies. The preliminary results obtained in patients should be informative to move forward the field of cancer metabolism-based therapeutics, and to discover whether the high expectations in these type of drugs might be justified.
